# Potent and selective inhibitors for M32 metallocarboxypeptidases identified from high-throughput screening of anti-kinetoplastid chemical boxes

**DOI:** 10.1371/journal.pntd.0007560

**Published:** 2019-07-22

**Authors:** Emir Salas-Sarduy, Lionel Urán Landaburu, Adriana K. Carmona, Juan José Cazzulo, Fernán Agüero, Vanina E. Alvarez, Gabriela T. Niemirowicz

**Affiliations:** 1 Instituto de Investigaciones Biotecnológicas “Dr. Rodolfo Ugalde”–Universidad Nacional de San Martín–CONICET, San Martín, B1650HMP, Buenos Aires, Argentina; 2 Departamento de Biofísica, Universidade Federal de São Paulo, São Paulo, Brazil; University of Oklahoma, UNITED STATES

## Abstract

Enzymes of the M32 family are Zn-dependent metallocarboxypeptidases (MCPs) widely distributed among prokaryotic organisms and just a few eukaryotes including *Trypanosoma brucei* and *Trypanosoma cruzi*, the causative agents of sleeping sickness and Chagas disease, respectively. These enzymes are absent in humans and several functions have been proposed for trypanosomatid M32 MCPs. However, no synthetic inhibitors have been reported so far for these enzymes. Here, we present the identification of a set of inhibitors for *Tc*MCP-1 and *Tb*MCP-1 (two trypanosomatid M32 enzymes sharing 71% protein sequence identity) from the GlaxoSmithKline HAT and CHAGAS chemical boxes; two collections grouping 404 compounds with high antiparasitic potency, drug-likeness, structural diversity and scientific novelty. For this purpose, we adapted continuous fluorescent enzymatic assays to a medium-throughput format and carried out the screening of both collections, followed by the construction of dose-response curves for the most promising hits. As a result, 30 micromolar-range inhibitors were discovered for one or both enzymes. The best hit, TCMDC-143620, showed sub-micromolar affinity for *Tc*MCP-1, inhibited *Tb*MCP-1 in the low micromolar range and was inactive against angiotensin I-converting enzyme (ACE), a potential mammalian off-target structurally related to M32 MCPs. This is the first inhibitor reported for this family of MCPs and considering its potency and specificity, TCMDC-143620 seems to be a promissory starting point to develop more specific and potent chemical tools targeting M32 MCPs from trypanosomatid parasites.

## Author summary

In recent years, the pharmaceutical company GlaxoSmithKline announced the disclosure of small collections of antiparasitic compounds to facilitate research and drug development for three of the main Tropical Neglected Diseases- i.e. Human African Trypanosomiasis, Leishmaniasis and Chagas Disease. These collections include new chemical entities with potential novel mechanisms of action that are likely to be active against a wide variety of targets. Taking advantage of these open access molecules, we successfully set up medium-throughput screening assays to find the first inhibitors of two metallocarboxypeptidases of the M32 family, a group of proteolytic enzymes proposed to play several roles in the biology of trypanosomatids including peptide catabolism, maintenance of parasite adaptive fitness and hydrolysis of bioactive peptides from the human host.

## Introduction

Members of the *Trypanosomatidae* family comprise parasitic organisms that cause highly disabling and often fatal diseases in humans and animals. The species that are responsible for human infections are *Trypanosoma brucei*, which cause Human African trypanosomiasis (HAT), *Trypanosoma cruzi*, the etiological agent of Chagas disease (American trypanosomiasis), and *Leishmania* spp., which cause different forms of leishmaniasis. Together, these vector-borne diseases constitute a substantial public health problem for which there is not a satisfactory treatment [1]. Major side-effects, and in some cases low effectiveness, are common problems associated with existing therapy. This situation makes imperative the development of new chemotherapeutic options. In this context, new drugs based on unique aspects of parasite biology and biochemistry are of great interest, particularly in the case of emerging resistance to traditional treatments [2–4]. In this scenario, proteases have become popular targets as these enzymes play key functions in parasite biology; namely nutrition, cell cycle progression, invasion and pathogenesis, among others.

The M32 family of metallocarboxypeptidases (MCPs) contains a group of hydrolases, which although being broadly distributed among prokaryotic organisms, are only present in a few eukaryotes including some green algae and trypanosomatids [5]. This unique phylogenetic distribution, in particular the absence of M32 enzymes in metazoans, has been considered an attractive trait due to the high specificity/selectivity potential of this family for drug target development. Within the *Trypanosomatidae* family several conserved M32 MCPs have been characterized [5–10]. Nonetheless, the cellular or biological functions of these proteins are currently unknown, as well as their essentiality status. In *T*. *brucei*, the genome-wide study by Alsford *et al*. (2011) reported no significant lost-of-fitness after induction of *T*. *brucei* MCP-1 (*Tb*MCP-1) RNAi in bloodstream and procyclic stages, as well as in the differentiation from procyclic to bloodstream forms [11]. More recently, however, it has been shown that *Tb*MCP-1 null mutant strains display extended doubling times in culture, suggesting that this enzyme might contribute to the adaptive fitness of the bloodstream form [12]. On the basis of their biochemical properties and stage-specific expression, the *L*. *major* M32 carboxypeptidase has been implicated in the catabolism of peptides and proteins to single amino acids required for protein synthesis [7]. The restricted substrate preference of *T*. *cruzi* MCP-1 (*Tc*MCP-1), plus its strong structural similarity to angiotensin I-converting enzyme (ACE), neurolysin and thimet oligopeptidase [8], have also pointed out a possible regulatory role of this family in the metabolism of small peptides. In fact, it has been shown that *Tc*MCP-1 can produce des-Arg9-bradykinin [6], a peptide that promotes the process of cell invasion through B1 receptors by the *T*. *cruzi* trypomastigotes [13]. In this sense, two reports have suggested that M32 peptidases are secreted by trypanosomatids [14, 15], a fact that is in agreement with this hypothesis. In the current scenario, the availability of selective small-molecule modulators of M32 MCPs activity would be of great value to ask mechanistic and phenotypic questions in both biochemical and cell-based studies. However, no inhibitors have been reported to date for these enzymes or other members of this family.

Recently, a diverse collection of ~ 1.8 million compounds from the proprietary library of GlaxoSmithKline (GSK) has been run through whole-cell phenotypic screens against *L*. *donovani*, *T*. *cruzi* and *T*. *brucei*. As a result, three anti-kinetoplastid chemical boxes of ~200 compounds each were assembled and open sourced [16]. The guiding design criteria for these molecule sets were chosen to include structures from different chemical families that are likely to be active against a wide variety of targets. By taking advantage of this diversity, we identified the first inhibitors of the M32 family of MCPs within the GSK HAT and CHAGAS chemical boxes. As model enzymes of the M32 family we employed *Tc*MCP-1 and *Tb*MCP-1, which have similar basic amino acid preference at the P1´ position and share 71% of protein sequence identity [5, 6].

## Results

### Development of continuous metallocarboxypeptidase assays

**Fig 1:**
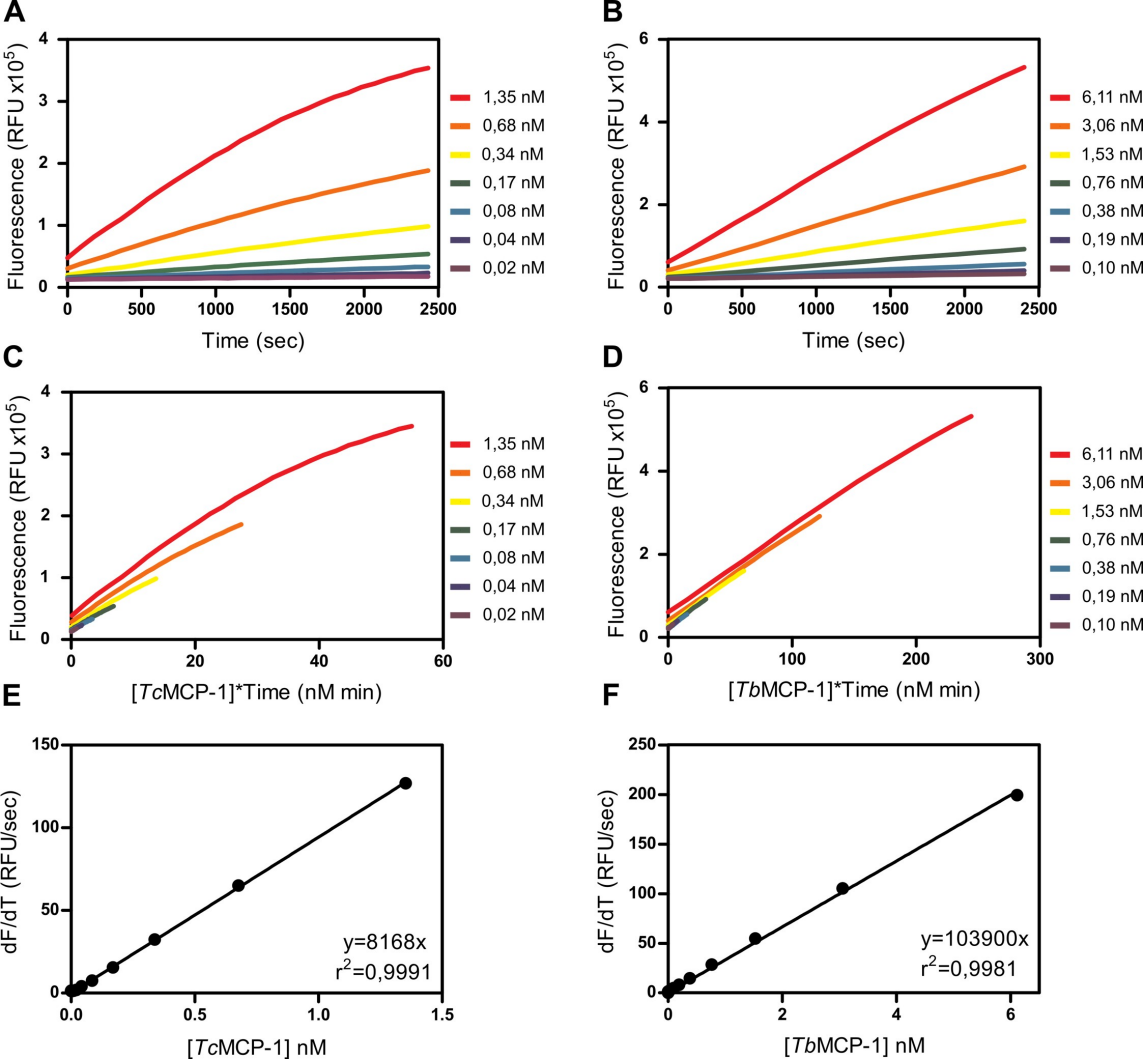
Continuous fluorogenic assays for recombinant MCPs. (A) Kinetic progression curves for different *Tc*MCP-1 concentrations at a fixed Abz-LKFK(Dnp)-OH dose (1,25 μM). (B) The activity of 2-fold dilutions of *Tb*MCP-1 was analyzed with Abz-RFFK(Dnp)-OH as substrate (4,8 μM). For both enzymes, working dilutions were selected from those that showed linear kinetics for more than 40 minutes. (C, D) Selwyn test for different *Tc*MCP-1 and *Tb*MCP-1 concentrations. In both cases, the global fitting of experimental data from different enzyme dilutions to a unique curve was good, indicating that enzymes remained stable during the whole assay. To facilitate observation, curves were slightly displaced from each other in the Y axis. (E, F) Curve of V_0_ vs. [E]_0_ for *Tc*MCP-1 and *Tb*MCP-1 respectively. In both cases, the expected linear behavior was observed.

To evaluate compounds in the HAT and CHAGAS chemical boxes, we devised a continuous assay for each MCP, based on FRET (fluorescence resonance energy transfer) peptides. We carried out the optimization process in 384 well plates, the same format used for the screening of the compound collections. For the selection of the most suitable substrate for the HTS assay, we initially assayed six FRET peptides against both enzymes. These were recently designed considering subsite preferences (P1´-P4) of *Tc*MCP-1 and *Tb*MCP-1 [12]. However, because no peptide was completely satisfactory for both enzymes, we selected independent substrates, Abz-LKFK(Dnp)-OH and Abz-RFFK(Dnp)-OH, for *Tc*MCP-1 and *Tb*MCP-1 assays, respectively. After substrate selection, a convenient enzyme concentration in the assay was determined through the activity of 2-fold dilutions of *Tc*MCP-1 and *Tb*MCP-1 at a fixed substrate concentration (Fig 1A and 1B). Moreover, the Selwyn test [17] revealed no enzyme inactivation under the conditions tested (Fig 1C and 1D). Thus, for a wide range of enzyme concentrations (for both MCPs), the V_0_ vs. [E]_0_ curves showed a linear behavior (Fig 1E and 1F). In particular, for [*Tc*MCP-1]_0_ < 0,34 nM and [*Tb*MCP-1]_0_ < 1,53 nM, the rate of the substrate hydrolysis remained constant for at least 40 minutes, a suitable time to perform the screening (Fig 1A and 1B).

The best balance between *Tc*MCP-1 activity on Abz-LKFK(Dnp)-OH substrate (estimated as dF/dt) and the time over which the reaction displayed linear kinetics was achieved at [*Tc*MCP-1]_0_ = 0,17 nM. Under these conditions, the enzyme showed the typical hyperbolic behavior predicted by the Michaelis-Menten equation (Hill coefficient = 1,06) and an estimated *K*_M_ value of 2,23 ± 0,28 μM (Fig A in S1 Text). Similarly, when the *Tb*MCP-1 concentration was fixed at 1,25 nM we obtained a *K*_M_ value on Abz-RFFK(Dnp)-OH substrate of 0,37 ± 0,06 μM (Hill coefficient = 1,03) (Fig A in S1 Text). To afford the best opportunity to find compounds with different inhibition modalities, we decided to employ balanced assay conditions (i.e. *K*_M_/[S] = 1)[18]. Using these conditions, preliminary characterization experiments of both optimized assays showed good general performance, with a dynamic range (μ^C+^—μ^C-^) higher than 15 RFU/sec, a μ^C+^/μ^C-^ ratio ≥ 50, good reproducibility (VC < 5%) and a Z´ factor value in the range 0,6–0,8.

### Primary screening of HAT and CHAGAS chemical boxes

Using the same lot of substrate and enzyme, the 404 compounds present in the HAT and CHAGAS chemical boxes were screened at a single fixed dose (25 μM). Each plate included 24 positive and negative controls, plus 16 wells containing 31,25 mM EDTA (inhibition control) alternately located in columns 11, 12, 23 and 24. In general, for each MCP, both plates presented highly similar Z´ scores although best values were obtained for the *Tb*MCP-1 assay presumably due to the lower background signal of the Abz-RFFK(Dnp)-OH substrate. To avoid the interference of highly fluorescent compounds, an auto-fluorescence cut-off value equal to 2x10^5^ RFU was used to accept or discard a molecule from the subsequent analysis. Using this limit, ~19% of the compounds were eliminated for *Tc*MCP-1 and *Tb*MCP-1 assays. Statistics are summarized in Table 1.

**Table 1:** Statistics for the plates during primary screening.

	*Tc*MCP-1				*Tb*MCP-1			
	Plate 1		Plate 2		Plate 1		Plate 2	
Compounds (*n*)	320		84		320		84	
	Mean	SD	Mean	SD	Mean	SD	Mean	SD
Enzyme control (C^+^) (RFU/sec)	19,12	1,60	20,19	2,14	16,80	0,57	21,23	0,57
Substrate control (C^-^) (RFU/sec)	2,98	0,19	3,63	0,24	0,01	0,20	-0,12	0,38
EDTA control (RFU/sec)	1,28	0,24	2,32	0,34	0,13	0,20	0,39	0,11
Z´ factor	0,67		0,57		0,86		0,87	

*Tb*MCP-1 and *Tc*MCP-1 activities were assayed fluorometrically with Abz-RFFK(Dnp)-OH and Abz-LKFK(Dnp)-OH substrates, respectively, in 100 mM MOPS pH 7,2 containing 0,01% Triton X-100 (C^+^). Final substrate concentration was set to a value *K*_M_ /[S] ~ 1. Additionally, 24 negative or substrate controls (no enzyme added, C^-^) plus 16 inhibition controls (EDTA final concentration 31,25 mM) were included in each plate. Z factor was calculated as in [19].

As shown in Table 2, if we consider a cut-off value ≤ 3 standard deviations from the control mean (μ^c+^ - 3σ^c+^), 70 and 132 inhibitory molecules were retrieved for *Tc*MCP-1 and *Tb*MCP-1, respectively. To reduce the number of resultant hits, we explored other two thresholds focusing only in outliers: i) those compounds showing slopes >3σ standard deviations above the average of all slopes in the plate (control independent) and ii) those compounds showing an inhibition percentage >3σ standard deviations above the average for the plate (control dependent). Interestingly, both criteria retrieved exactly the same list of compounds for *Tc*MCP-1 (n = 5) while for *Tb*MCP-1 the intersection between this two groups was lower (2 out of 4 compounds).

**Table 2:** Primary screening results.

	*Tc*MCP-1		*Tb*MCP-1	
	Plate 1	Plate 2	Plate 1	Plate 2
Compounds (*n*)	320	84	320	84
Analyzed compounds (*n*)*	256	72	254	71
1**	51	19	92	40
2**	4	1	2	2
3**	4	1	0	2
40% inhibition	19	4	22	5

(*) Highly fluorescent compounds were discarded from the analysis.

(**) Different hit selection criteria were applied to both HAT and CHAGAS boxes. 1) Compounds showing an inhibition ≤ three standard deviation from control mean. 2) Compounds showing slopes >3σ standard deviations above the average of all slopes in the plate and 3) those compounds showing a percent inhibition >3σ standard deviations above the average for the plate.

### Secondary screening

**Fig 2:**
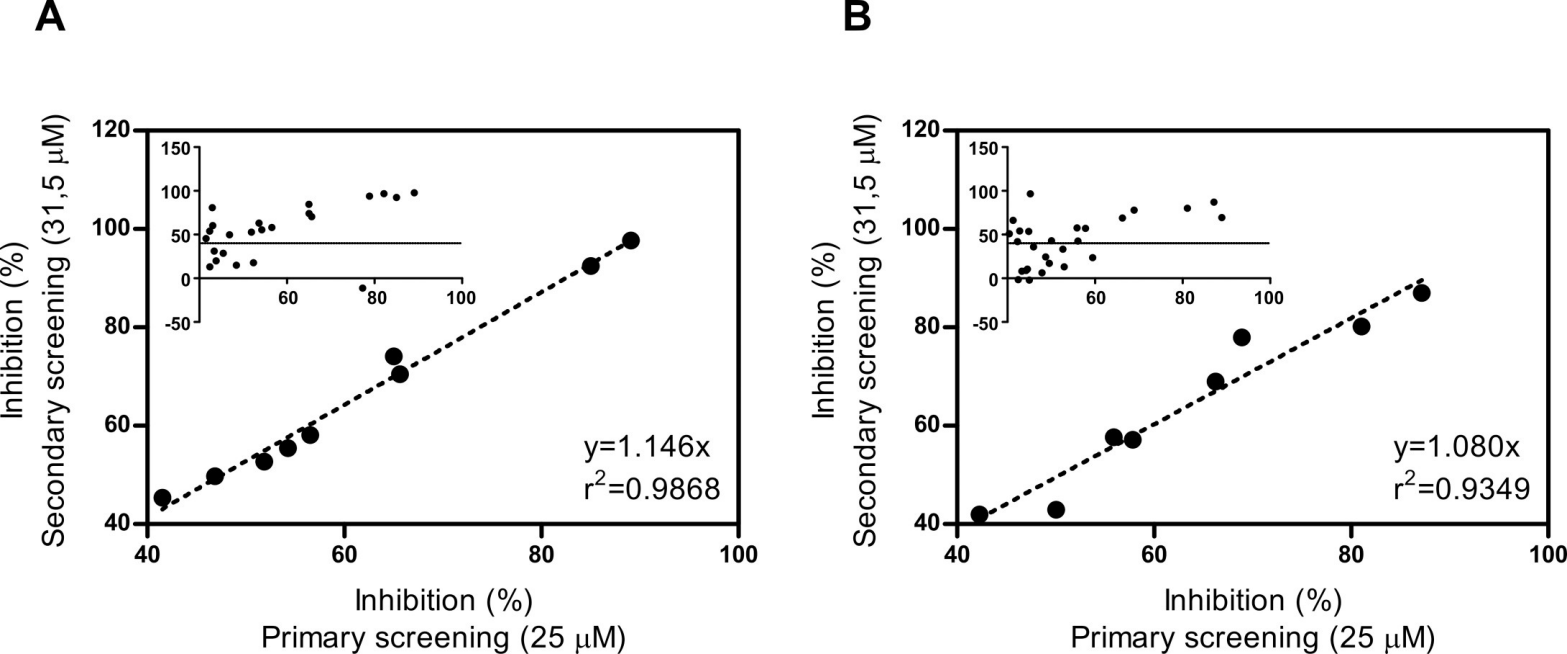
Correlation between the inhibition percentages in the primary and secondary screenings for the most reproducible compounds. As both screening rounds were performed without technical replicates (see Material and Methods), we introduced this analysis to assess data consistency. The analysis was performed using the 25μM and 31,5μM data points from the primary and secondary screenings, respectively. The main panel shows correlation of the most reproducible hits, whereas the insets show the correlation analysis for all the hit compounds at indicated concentrations. (A) *Tc*MCP-1 (B) *Tb*MCP-1.

In the secondary screening we decided to include all compounds that showed ≥ 40% of inhibition (*Tc*MCP-1: 23 compounds; *Tb*MCP-1: 27 compounds). To estimate IC_50_ for the resulting hits, two-fold serial dilutions, ranging from 7,5 pM to 62,5 μM, were analyzed against both recombinant MCPs using identical assay conditions as in the primary screening. Prior to the analysis of the complete dataset, we examined whether there was a correlation between the inhibition percentages in the primary (compound concentration 25 μM) and secondary screening, using only the data corresponding to a compound concentration of 31,5 μM. This was important to assess consistency of data, as both screening rounds were performed without technical replicates due to limitation of compound stocks. For *Tc*MCP-1, 9 compounds presented similar behavior in both screenings (correlation coefficient r^2^ = 0,9868; slope = 1,146) (Fig 2A) whereas 7 molecules failed to reach ≥ 40% of inhibition threshold (n = 6) or displayed no inhibition (n = 1) (correlation coefficient r^2^ = -0,518; slope = 0,2595). Additionally, 7 compounds performed better in the secondary screening (correlation coefficient r^2^ = 0,5156; slope = 1,2749). For the *T*. *brucei* enzyme, consistent results in both assays were achieved only by 8 compounds (correlation coefficient r^2^ = 0,9349; slope = 1,080) (Fig 2B). About 45% of the samples did not repeat the ≥ 40% of inhibition criterium (n = 10) or did not inhibit (n = 2) *Tb*MCP-1 (correlation coefficient r^2^ = 0,1163; slope = 0,3173). Finally, another 7 molecules performed better in the secondary screening than in the first round. Despite the observed round to round discrepancies (Table A in S1 Text), we decided to continue curve analysis for all the compounds, with the exception of the three that showed no inhibition at 31,5 μM during secondary screening.

**Fig 3:**
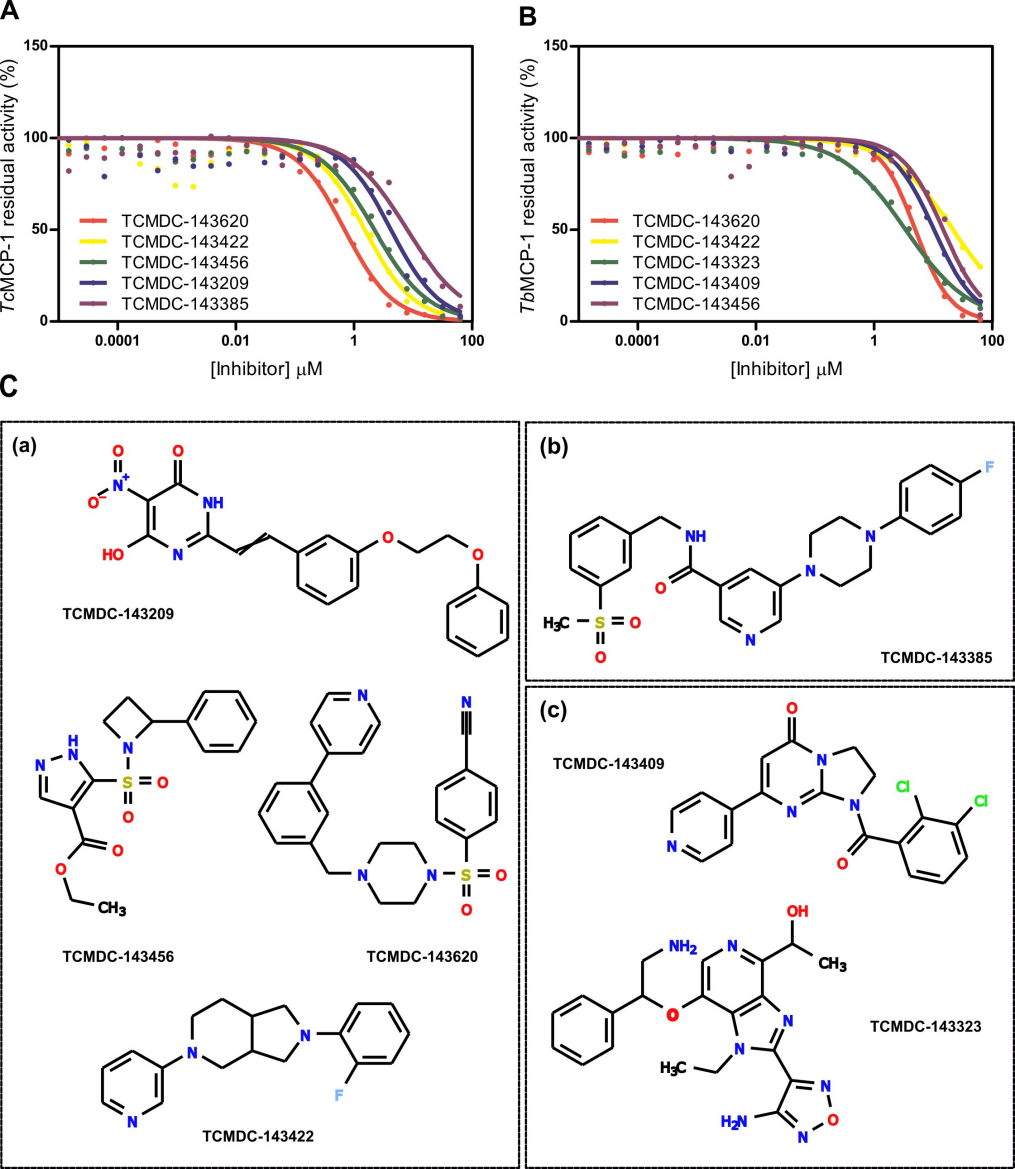
Dose-response curves and structures of top-five inhibitors identified for each MCPs. To estimate the potency of the inhibitory activity, enzymes were incubated with different concentrations (ranging from 7,5 pM to 62,5 μM) of the selected compounds and the inhibition percentages determined for each condition as indicated in Material and Methods. For each compound, solid line represents the best fit of four-parameter Hill equation to experimental data (closed circles). (A) Dose-response curves corresponding to the most potent *Tc*MCP-1 inhibitors. (B) Equivalent analysis for the top-five *Tb*MCP-1 inhibitors. (C) Structure and identifiers corresponding to the most potent hit compounds identified for both enzymes. Subgroup (a) contains those molecules that inhibited both MCPs. Subgroup (b) is formed by TCMDC-133485, which selectively acts on *Tc*MCP-1 whereas subgroup (c) includes *Tb*MCP-1 specific inhibitors.

For *Tc*MCP-1, five compounds (TCMDC-143620, TCMDC-143422, TCMDC-143456, TCMDC-143209 and TCMDC-143385) showed an IC_50_ value ≤ 10 μM (Fig 3A and Table 3). In good agreement, the four more potent molecules (TCMDC-143620, TCMDC-143422, TCMDC-143456 and TCMDC-143209) also inhibited the *T*. *brucei* enzyme (Table 3). Compounds TCMDC-143385 and TCMDC-143172 (which display an IC_50_ ~10 μM for *Tc*MCP-1) did not reach the 40% inhibition threshold in the *Tb*MCP-1 primary screening and were left out from the secondary analysis. Other potent molecules, namely TCMDC-143409 and TCMDC-143323 were specific inhibitors of *T*. *brucei* enzyme or produced little inhibition on *Tc*MCP-1 (< 30%) (Fig 3B and Table 3). The structure of the top-five inhibitors for each enzyme is shown in Fig 3C.

**Table 3:** IC_50_ values and Hill slopes for identified hits.

Compound	Chemical Box	*Tc*MCP-1				*Tb*MCP-1		
		IC_50_ (μM)	Hill Slope	R square		IC_50_ (μM)	Hill Slope	R square
TCMDC-143620	CHAGAS	0,6939	-1,06	0,9821		4,989	-1,461	0,9674
TCMDC-143422	CHAGAS	1,52	-1,038	0,8644		**10,46**	**-1,206**	**0,8407**
TCMDC-143456	HAT	**2,206**	**-0,9597**	**0,9609**		**14,74**	**-1,247**	**0,9121**
TCMDC-143209	CHAGAS	**4,182**	**-1,019**	**0,8086**		**28,34**	**-1,2405**	**0,9851**
TCMDC-143385	CHAGAS	9,473	-0,9614	0,838		-		
TCMDC-143172	HAT	11,21	-2,825	0,9751		-		
TCMDC-143513	HAT	**13,52**	**-0,9734**	**0,8486**		28,33	-0,8371	0,9523
TCMDC-143551	HAT	**13,84**	**-1,834**	**0,4295**		**34,51**	**-1,562**	**0,5659**
TCMDC-143462	HAT	15,48	-0,9436	0,8296		-		
TCMDC-143382	HAT	20,59	-0,7277	0,861		**22,55**	**-0,7258**	**0,9649**
TCMDC-143515	HAT	**21,83**	**-1,149**	**0,7376**		31,71	-0,8447	0,9624
TCMDC-143432	CHAGAS	26,26	-0,6129	0,9382		-		
TCMDC-143242	HAT	**26,5**	**-1,237**	**0,851**		-		
TCMDC-143592	CHAGAS	**27,91**	**-0,8919**	**0,8208**		-		
TCMDC-143408	CHAGAS	**32,86**	**-1,153**	**0,9473**		-		
TCMDC-143496	HAT	**34,9**	**-0,822**	**0,6521**		-		
TCMDC-143071	CHAGAS	38,91	-1,235	0,8373		-		
TCMDC-143263	HAT	40,74	-0,9946	0,8174		-		
TCMDC-143543	HAT	>60						
TCMDC-143323	HAT	-				**3,938**	**-0,894**	**0,9353**
TCMDC-143409	CHAGAS	-				10,42	-1,2409	0,9815
TCMDC-143191	CHAGAS	-				16,11	-1,267	0,977
TCMDC-143645	HAT	-				20,79	-1,127	0,9238
TCMDC-143143	CHAGAS	-				**21,78**	**-1,466**	**0,9195**
TCMDC-143332	CHAGAS	-				23,27	-1,063	0,9614
TCMDC-143158	HAT	-				27,64	-1,014	0,8757
TCMDC-143254	HAT	-				43,43	-0,8163	0,768
TCMDC-143265	HAT	-				44,2	-2,036	0,7989
TCMDC-143454	HAT	-				>60		
TCMDC-143187	CHAGAS	-				>60		

Compounds that presented similar behavior in both primary and secondary screening (<15% variation between both assays) are highlighted in grey.

### Lead compounds have low structural redundancy

**Fig 4:**
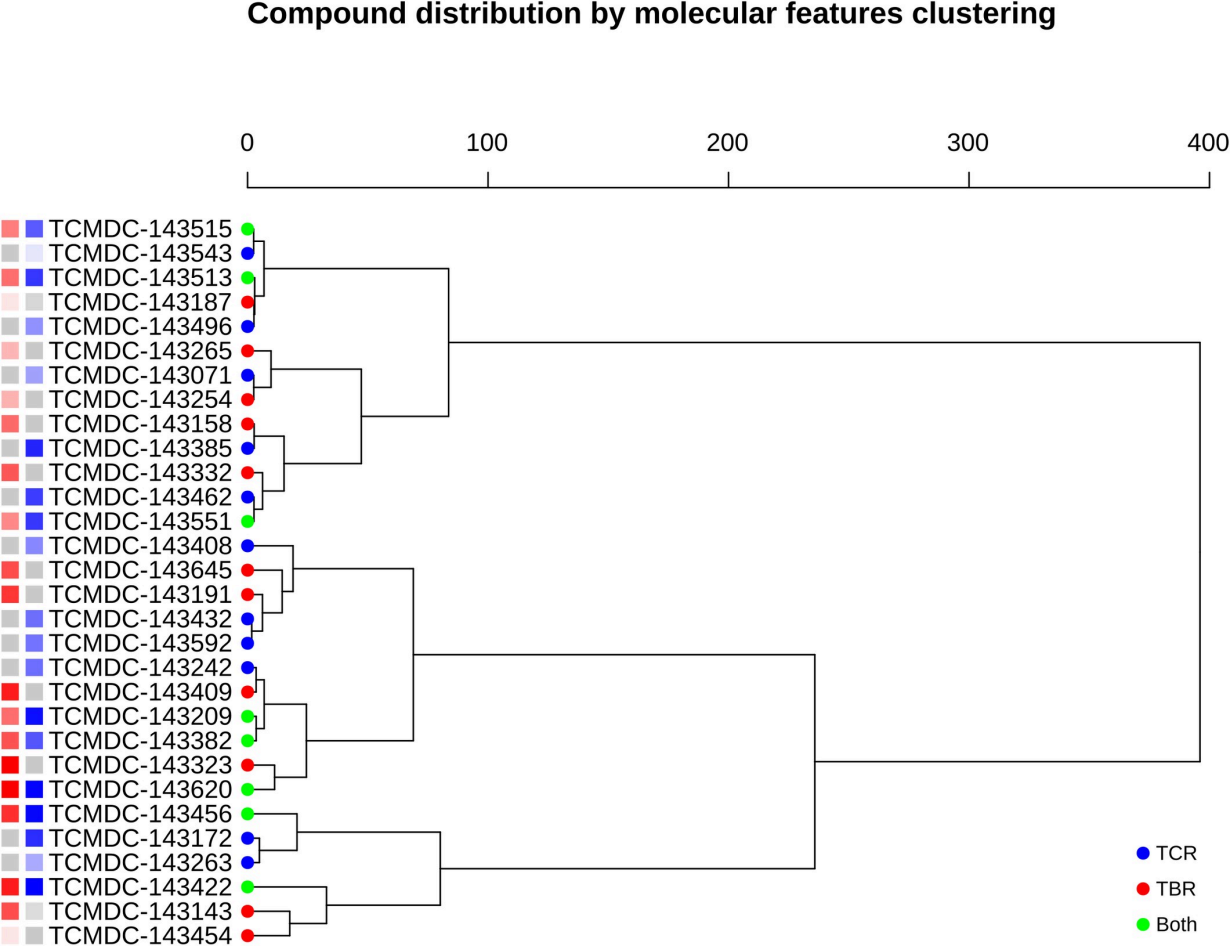
A dendrogram representing compound clustering using molecular features, and summarizing the activity distribution among tested MCPs. Squares next to the names give an idea of IC_50_ observed: the brighter the color, the lower the IC_50_. Red squares for *Tb*MCP-1, blue squares for *Tc*MCP-1, grey squares for non-active in *Tb*MCP-1/*Tc*MCP-1, accordingly.

To first assess the possibility that these lead compounds have shared structural features that help explain their bioactivity profile, we performed three different clustering strategies: one using Tanimoto similarity (Fig B in S1 Text), one based on shared substructures (overlap of *Maximum Common Subgraphs*, MCS) (Fig C in S1 Text), and the third one based on shared physicochemical properties (Fig 4). Whereas the Tanimoto clustering was expected to be inconclusive based on the premises used to assemble the chemical boxes (one or two putative chemotypes per box [16]); the clustering based on physicochemical properties also showed no significant correlation between these properties and the observed IC_50_s. Similarly, MCS clustering provided no insights into candidate substructures guiding the activity or specificity of the compounds against each enzyme. In all three strategies, the clusters not only group up dissimilar potencies, but also mix compounds with different enzyme specificity.

### Most compounds have at least one Zinc-biding group

To determine the number and type of Zinc-binding groups (ZBGs) among the compound leads, an MCS analysis was performed using an *ad hoc* curated [20, 21] database of ZBGs. From a total of 48 groups available in the database, only six of them were found among 24 of the 30 lead compounds: pyridine (14 compounds), sulfonamide (7 compounds), imidazole (4 compounds), pyrazole (3 compounds), diol (1 compound) and hydrazide (1 compound). The majority of compounds (24 out of 30) presented at least one ZBG in the structure. More specifically, 15 with a single group and 9 with two groups were found. All compounds and their corresponding ZBGs have been summarized in Fig D in S1 Text.

### MCP inhibitors are specific

**Fig 5:**
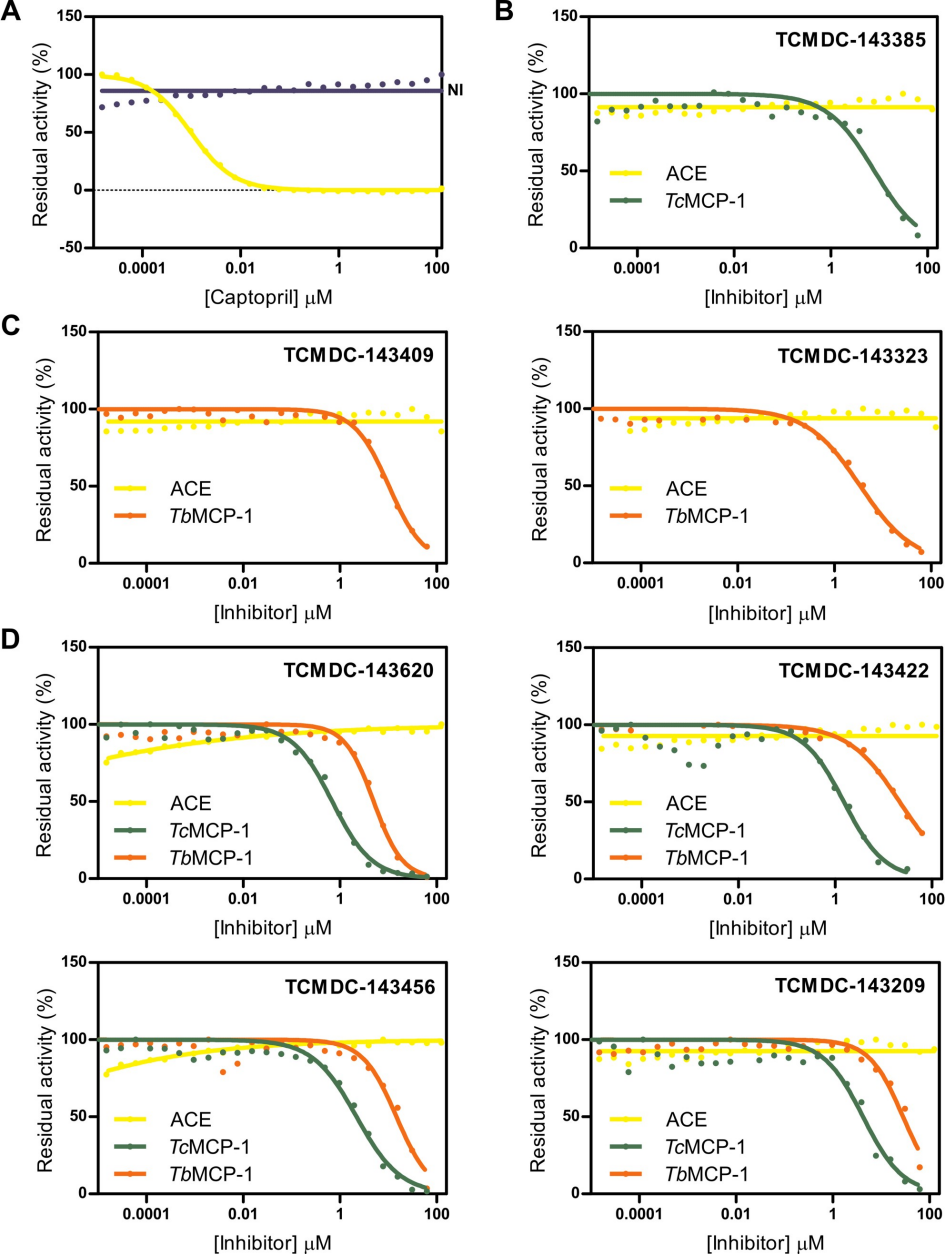
Dose-response curves for ACE. (A) Purified rabbit lung ACE was assayed at 37°C in 0,1 M Tris-HCl pH 7,0 buffer containing 50 mM NaCl, 10 mM ZnCl_2_ and 0,01% Triton X-100. Captopril, a potent competitive ACE inhibitor, was included as a positive inhibition control (IC_50_~ 1 nM). NI, no inhibitor added. (B, C) Data corresponding to *Tc*MCP-1 and *Tb*MCP-1 specific inhibitors, respectively. (D) Dose-response curves corresponding to those molecules that inhibited both MCPs. In all cases, solid lines represent the best fit of four-parameter Hill equation to experimental data (closed circles). Yellow, dark-green and orange colors were used for ACE, *Tc*MCP-1 and *Tb*MCP-1, respectively.

Considering the abundance of ZBGs and heteroatom-containing moieties in the hits, we evaluated the possibility of a nonspecific mechanism of inhibition (involving metal chelation) for the top-five inhibitors identified in the screening for each enzyme. Because M32 MCPs show a strong topological similarity with ACE [22], we chose this enzyme to estimate the IC_50_ value for each molecule. As done for the MCPs essays, ACE activity was analyzed employing a FRET substrate, Abz-FRK(Dnp)P-OH, at a concentration equal to the apparent *K*_M_ of the enzyme ~3 μM [23]. Experiment set up is summarized in Figs E and F in S1 Text. For comparative purposes, captopril, a potent competitive ACE inhibitor, was included in the analysis (IC_50_ ~1 nM) (Fig 5A). Under these conditions, no inhibition could be detected for any of the compounds evaluated, thus suggesting that these molecules are not promiscuous metallocarboxypeptidase inhibitors (Fig 5B, 5C and 5D) but are instead specific inhibitors of M32 MCPs.

## Discussion

M32 MCPs have an unusual phylogenetic distribution (with trypanosomatids being among the few eukaryotic genomes encoding these enzymes). Hence M32 MCPs from parasites arose naturally as interesting candidates for drug target development. Furthermore, the current lack of knowledge about the cellular and/or physiological role(s) of these enzymes makes the identification of potent inhibitors a task of great significance, as these compounds may be used as molecular probes to potentially identify natural substrates, to recognize the specific pathways in which they are involved or, hopefully, to perform their chemical validation as drug targets. In this work, we describe the first drug-like inhibitors of *Tc*MCP-1 and *Tb*MCP-1, two closely related MCPs from the human pathogens *T*. *cruzi* and *T*. *brucei*, respectively. Our starting point were the GSK HAT and CHAGAS boxes, two small collections containing non-redundant, chemically diverse and highly bioactive compounds [16], which could facilitate future optimization efforts.

Although we initially aimed for a common assay for both MCPs, we soon realized that the use of different FRET substrates for each enzyme resulted in better general performance of the individual assays (considering signal robustness, temporal duration of linear kinetics, dynamic range, μ^C+^/μ^C-^ ratio and Z´ factor). Surprisingly, the substrates that resulted most suitable for the developed HTS assays were not, in any case, those that showed the best values of k_cat_, *K*_M_ and k_cat_/*K*_M_ in their previous kinetic characterization [12]. Although different assays were used to screen these collections, we were able to find specific inhibitors for both enzymes, and perhaps more important, mutual inhibitors; suggesting the consistency of inter-assay results. Of note, specific inhibitors for each enzyme were distributed evenly among HAT and CHAGAS boxes with no apparent bias. This fact confirms the importance of not circumscribing the search to just the pathogen-specific box, but instead to widen the search to all the boxes available, as previously observed for *T*. *cruzi* cysteine peptidase cruzipain [24].

Due to the limited amount of compound stocks, we decided to implement the screening of chemical boxes in singlet, with primary evaluation of all compounds at a fixed dose and further dose-response analysis of unconfirmed hits in a secondary screening. As expected, given the error-prone nature of the single-well (single dose, single replicate) measurements used in primary screening, significant discrepancies in inhibition were observed for some compounds in comparison to secondary dose-response evaluation. These discrepancies are common and may be due to a variety of factors [25]. Besides intrinsic compound-specific and experimental data variability [26], these factors may include solubility issues (given that in primary and secondary screenings both the final concentration and serial-dilution protocol were different), differential stability of compounds in stock (10 mM) and working (2 mM) solutions [27], unintended absorption of the compounds to different containing materials during storage, moderate dose-dependent quenching effects of compounds on fluorescence readouts, among others [28]. In addition, although we included 0,01% Triton X-100 in assay buffer, compound-specific aggregate formation was not tested and thus, cannot be dismissed.

As mentioned, we identified in this work eight molecules able to inhibit both MCPs. These mutual inhibitors came from both boxes in similar numbers, as previously noted for enzyme-specific compounds. Interestingly, in all cases they were more potent inhibitors of *Tc*MCP-1, for reasons that are as yet unclear. Importantly, four of these compounds proved to be inactive on ACE, a Zinc-dipeptidyl carboxypeptidase involved in various physiological and physiopathological conditions in mammals [29] which shows significant structural similarity to M32 enzymes [22, 30]. This fact strongly suggests that despite the structural resemblance and the small number of compounds tested here, the identification of inhibitors with high selectivity for trypanosomatid M32 MCPs over ACE can be achieved, a point in favor to the specific druggability of these enzymes.

The identified inhibitors display high structural diversity, with many showing only marginal similarity to the other hits, hence representing different structural clusters and presumably, different inhibitory scaffolds. In this regard, the presence of “unpaired” hits is not surprising, considering that no more than two members of the same structural cluster were included per box during collection assembly [16] and that “twin” compounds might well not pass the activity or auto-fluorescence filters included in this work. Among the identified inhibitors, only TCMDC-143265 and TCMDC-143551 share similar core structures, thus probably populating the same cluster and sharing a common active scaffold. A significant part of both molecules is identical and adopts the same spatial conformation (Fig G in S1 Text), with the largest differences located around the benzamide ring. Besides the obvious differences in the length and position of sulfonamide substituents, the chlorine substitution in position 2 imposes a ~90° rotation of the benzamide ring in TCMDC-143265 compared to TCMDC-143551, where all ring systems are almost coplanar. Interestingly, these structural differences seem to dictate the selectivity toward *Tc*MCP-1, as TCMDC-143551 inhibits both enzymes whereas TCMDC-143265 is specific for *Tb*MCP-1. Even for this pair of compounds, there is no evident substructure responsible for M32 MCPs bioactivity; though this is probably a biased observation due to the lack of well-defined structural features for M32 MCPs inhibitors.

Although the crystallographic structure of *Tc*MCP-1 has been determined [8] and subsite specificity have been explored for both enzymes using FRET substrate libraries [12] and mutagenesis [6, 8], little is yet known about how substrates are accommodated into the catalytic groove, which residues are key determinants of subsite specificity and the significance of the hinge-type movement between L and R domains in the stabilization of enzyme-substrate or enzyme-inhibitor complexes. With all these gaps to fill, it seems risky to speculate about the modes of interaction of these new inhibitors with *Tc*MCP-1 and *Tb*MCP-1. However, a presumptive explanation can be put forward. As in the case of many other metallopeptidase inhibitors, it is likely that inhibition of trypanosomatid M32 MCPs occurs throughout the perturbation of the coordination sphere of the catalytic metal ion (presumably Zn^2+^ in the case of *Tc*MCP-1 and *Tb*MCP-1, by extension from other M32 enzymes [31]). Typically, synthetic metallopeptidase inhibitors achieve preliminary affinity and target selectivity through the formation of stabilizing interactions with specific residues within the active site; while a ZBG is responsible for metal chelation, enhancing binding affinity, modulating selectivity and disrupting catalytic activity [32]. For the majority of the inhibitors presented here, it was possible to identify typical ZBG or at least, heteroatom-containing groups able to establish a coordinative bond with a Zn^2+^ ion (Fig D in S1 Text). For those compounds, an inhibition mechanism like the one described above is possible. For other molecules not having a Zn-coordinating group, the most plausible explanation is that inhibition occurs as a result of the prevention of substrate binding by the partial occupancy or the deformation of the catalytic cleft by the inhibitor molecule, as previously observed for Non-Zinc-Binding inhibitors of other metallopeptidases [33].

The vast majority of the hits identified here inhibit one or both MCPs in the micromolar range, with only a few of them showing potencies <10 μM. Outstandingly, TCMDC-143620 inhibits *Tc*MCP-1 in the sub-micromolar range (it also inhibits *Tb*MCP-1, but with potency ~7-fold lower). This is the most potent inhibitor described so far for an enzyme of the M32 family and seems a promising candidate for further structure-based optimization. The unusually high flexibility of the M32 MCPs around the active site [31, 34] prevented us to use a docking approach to get insights of the binding mode of this compound within *Tc*MCP-1 and *Tb*MCP-1 catalytic clefts. However, the TCMDC-143620 molecule seems able to form a variety of stabilizing interactions. These may include hydrophobic and electrostatic interactions, hydrogen bonding and the coordination to the metal ion through the pyridine ring. In addition, the presence of a central sulfonamide group and a distal nitrile group add further interaction possibilities to this molecule. For example, the sulfonamide group has been extensively incorporated into metallopeptidase inhibitors due to its ability to improve the enzyme-inhibitor binding by different mechanisms. These mechanisms include: i) direct formation of hydrogen bonds to the enzyme backbone, ii) properly redirection of bulky groups into enzyme pockets by inducing a twist in the structure of the inhibitor molecule and iii) even cooperate with other chelating groups in the coordination of the catalytic metal ion [35]. Similarly, the nitrile group in TCMDC-143620 can establish polar interactions, hydrogen bonds or react with serine or cysteine side chains to form covalent adducts which would greatly stabilize inhibitor binding [36]. Interestingly, the nitrile group is also able to form coordinative bonds with a variety of metal ions including Co^2+^, Mn^2+^, Fe^3+^, Cu^2+^ and Zn^2+^ [37]. Thus, a possible role of this group in the direct coordination of the catalytic metal ion cannot be discarded at present. The determination of the crystallographic structure of *Tc*MCP-1 or *Tb*MCP-1 in complex with TCMDC-143620 would provide a definitive answer to these questions as well as important clues to undertake the future lead-optimization of this hit.

A preliminary analysis of the bioactivity profile of TCMDC-143620 (https://pubchem.ncbi.nlm.nih.gov/compound/91800813) indicates that it shows potent activity against *T*. *cruzi* in culture and only moderate but measurable activity on *T*. *brucei* and *L*. *donovani*. Also, this compound exhibits moderate cytotoxicity on mammalian cell NIH 3T3 (IC_50_ = 13 μM) but resulted inactive on HepG2 (IC_50_ > 100 μM). Considering target-specific assays; this compound has a single bioactivity report. TCMDC-143620 was found to be a potent inhibitor (IC_50_ = 79 nM) of *T*. *cruzi* sterol 14-α demethylase (CYP51) enzyme, which is involved in the ergosterol biosynthesis pathway and was considered until recent years as a promissory therapeutic target for Chagas disease [38, 39]. The inhibition of this target is probably the cause of its reported anti-*T*. *cruzi* activity. This might also explain, at least partially, the moderate cytotoxic and anti-*T*. *brucei* and *L*. *donovani* activities reported for this compound, considering the global similarities of enzymes within CYP51 family [40, 41]. Although involved in other studies as part of the GSK CHAGAS Box [42], no further information is currently available from the evaluation of TCMDC-143620 against other molecular targets, except for our previous cruzipain study [24] where it was found to be inactive (~7,5% of cruzipain inhibition at 25 μM). A complete profile of the off-target activity of TCMDC-143620 would be critical for future optimization efforts in order to achieve a suitable M32 MCPs probe from this compound.

In summary, 30 micromolar-range inhibitors, presenting both high structural diversity and novelty, have been discovered for *Tc*MCP-1 and/or *Tb*MCP-1 by using continuous, fluorescent-based and HTS-capable enzymatic assays. The best hit shows sub-micromolar affinity for *Tc*MCP-1, inhibits *Tb*MCP-1 in the low micromolar range and, like other potent hits, is inactive on ACE. Considering its potency and specificity, this molecule seems to be a promissory starting point to develop more specific and potent tools to expand our understanding of the biochemistry and biological role(s) of M32 MCPs from trypanosomatid parasites and, hopefully, to assess in a near future their value as drug targets.

## Materials and methods

### Reagents

Triton X-100, MOPS (3-(N-morpholino)propanesulfonic acid), DMSO, EDTA and captopril were purchased from Sigma-Aldrich. Substrates Abz-RFFK(Dnp)-OH and Abz-LKFK(Dnp)-OH were from GenScript (Piscataway, NJ, USA). Black solid bottom polystyrene Corning NBS 384-well plates were from Sigma-Aldrich (CLS3654-100EA).

### Enzymes

*Tc*MCP-1 (*MEROPS* ID: M32.003) and *Tb*MCP-1 were expressed as GST fusion proteins in *E*. *coli* BL21 (DE3) Codon Plus and purified as previously described [6, 8].

### Anti-kinetoplastid chemical boxes

The HAT and CHAGAS chemical boxes [16] were provided by GlaxoSmithKline. The collection comprised 404 compounds, prepared as 10 mM stock solutions in DMSO (10 μL each) and dispensed in 96 well plates. For primary screening, a working solution (final concentration of 2 mM) for each compound was prepared by 1/5 dilution in DMSO while 1 μL of the 10 mM stock solution was used for secondary screening of selected compounds, as previously described [24]. The final concentration of compounds tested in primary screening was 25 μM, while the compound concentrations assayed in secondary screening ranged from 7,5 pM to 62,5 μM.

### MCPs assays

*Tb*MCP-1 and *Tc*MCP-1 activities were assayed fluorometrically with Abz-RFFK(Dnp)-OH and Abz-LKFK(Dnp)-OH substrates, respectively, in 100 mM MOPS pH 7,2 containing 0,01% Triton X-100. Assays were performed in solid black 384-well plates (final reaction volume ~80 μL) and the hydrolysis of the K(Dnp)-OH group was monitored continuously at 30 °C with a Beckman Coulter DTX 880 Multimode Reader (Radnor, Pennsylvania, USA) using standard 320  nm excitation and 420 nm emission filter set.

For each MCP, final substrate concentration was set to a value *K*_M_ /[S] ~ 1. Optimal enzyme concentration was selected from 2-fold serial dilutions to match three criteria: (i) being linearly proportional to V_0_, (ii) display robust signal evolution at substrate concentration chosen and (iii) display linear kinetics for enough time to perform several reading cycles (at least 8 cycles, minimum time between cycles: 264 sec) through the 384-wells. In all cases, EDTA (final concentration 31,25 mM) was used as positive inhibition control.

### Primary screening

To perform the primary screening, 1 μL of each compound (2 mM in DMSO, final concentration in the assay: 25 μM), EDTA (500 mM, final concentration in the assay: 31,25 mM) were dispensed into 384-well Corning black solid-bottom assay plates. Then, 40 μL of 100 mM MOPS, 0,01% Triton X-100 pH 7,2 containing *Tb*MCP-1 (2,50 nM) or *Tc*MCP-1 (0,34 nM) were added to each well, plates were homogenized (30 seg, orbital, medium intensity) and each well subjected to a single autofluorescence read (ex/em  =  320/420 nm). Plates were incubated in darkness for 15 min at 30 °C and then 40 μL of Abz-RFFK(Dnp)-OH (4 μM) or Abz-LKFK(Dnp)-OH (0,8 μM) in assay buffer were added to each well to start the reaction. After homogenization (30 seg, orbital, medium intensity), the fluorescence of the Abz group (ortho-aminobenzoic acid) (ex/em  =  320/420 nm) was acquired kinetically for each well (8 read cycles, one cycle every 300 seconds). Considering our previous experiences, the auto-fluorescent cut-off was arbitrarily set at 2x10^5^ RFU to discard highly interfering compounds. All compounds were assayed in singlet (without replicates) due to the limited availability of stocks.

Raw screening measurements were used to determine the slope (dF/dt) of progression curves by linear regression for control and non-interfering compound wells. In the case of control-dependent hit selection criteria, percent inhibition percentage (%Inh) was calculated for each compound according to the following equation:

$$Inh=100∙\left[1-\frac{\left({\frac{dF}{dt}}^{WELL}-\mu^{C-}\right)}{\left(\mu^{C+}-\mu^{C-}\right)}\right]$$ (1)

where dF/dt^WELL^ represents the slope of each compound well and μ^C+^ and μ^C−^ the average of MCP (no-inhibition) and substrate (no-enzyme) controls, respectively.

### Secondary assay

Compounds selected from primary screening were re-tested in a dose-response manner (final concentration ranging from 7,5 to 62,5 μM) using identical assay conditions. To avoid any positional and/or association bias, we randomly defined the row position for each compound. One μL of compounds stock (10 mM in DMSO) and EDTA (31,25 mM) were added to the first well of column 1, followed by addition of 40 μL of 100 mM MOPS, 0,01% Triton X-100 pH 7,2 buffer. After addition of 20 μL of the same buffer to subsequent wells of the plate, 22 serial 2-fold dilutions were made horizontally. The last two positions of every row were used, alternatively, for C^+^ and C^−^ controls to reduce any positional and/or association bias. Then, 20 μL of activity buffer containing *Tb*MCP-1 or *Tc*MCP-1 were added to each well, except for those corresponding to C^−^; completed with 20 μL of activity buffer. After homogenization, 15 minutes of incubation at 30°C and autofluorescence measurement, the substrate (in activity buffer) was added to the previous mix. Data collection and processing were performed exactly as described above. Percentage of M32 MCPs residual activity was calculated for each condition according to the following equation:

$${\%Res.Act}^{MCP}=100∙\left[\frac{\left({\frac{dF}{dt}}^{WELL}-\mu^{C-}\right)}{\left(\mu^{C+}-\mu^{C-}\right)}\right]$$ (2)

where dF/dt^WELL^ represents the slope of each compound well and μ^C+^ and μ^C−^ the average of MCP (no-inhibition) and substrate (no-enzyme) controls, respectively. The IC_50_ and Hill slope parameters for each compound were estimated by fitting the four-parameter Hill equation to experimental data from dose-response curves using the GraphPad Prism program (version 5.03).

### ACE assay

Purified rabbit lung ACE (EC 3.4.15.1) was purchased from Sigma-Aldrich. Enzyme activity was assayed fluorimetrically with Abz-FRK(Dnp)P-OH (ex/em  =  320/420 nm) as substrate in buffer 0,1 M Tris-HCl, 50 mM NaCl, 10 mM ZnCl_2_, pH 7.0 containing 0,01% Triton X-100 as indicated in [23]. Selected compounds were tested in a dose-response manner (final concentration ranging from 7,5 pM to 62,5 μM) using identical assay conditions employed with both MCPs. Captopril (15 pM—125 μM) was used as inhibition control.

### Compound clustering

Three separate compound clustering routines were used. One of them derived from calculated or predicted molecular features, and the other two directly inferred from different distance metrics between compounds: one using Tanimoto similarity and another one using the overlap score calculated in a MCS (*Maximum Common Subgraph*) pipeline. The Tanimoto distance compound clustering was performed to rapidly find compound pairs, if available, within the leads. OpenBabel 2.4.1 [43] was used to export molecule MDLs from SMILES format, available from GSK chembox summary.

For Tanimoto clustering, the indexes were calculated using ChemFP 1.3 [44] with ob2fps bindings and simsearch -NxN as parameter. ChemFP results were parsed and analyzed using an *ad hoc* perl script, setting the distance (D) between compounds as D = 1—T_index_. The distance matrix was built using *melt* and *acast* from R Data table package [45].

To assess the MCS clustering, all compounds were imported into a R script using Chemminer [46] and further analyzed using fmcsR [47] for batch MCS calculations.

For the molecular feature clustering, a perl script was built to run XlogP3 v3.2.2 [48] through all lead compounds. Features used to build distance matrix, along with their corresponding values, can be found in Table 4. All clustering plots were achieved using the R base hierarchical clustering tool, hclust.

**Table 4:** Molecular features used to build compound clustering dendrogram.

NAME	MW	HBD	HBA	RB	RINGS	XLOGP
TCMDC-143071	416,3	1	3	6	4	3,18
TCMDC-143143	242,2	1	3	2	2	4,36
TCMDC-143158	443,3	1	3	6	6	1,46
TCMDC-143172	299,1	1	2	3	3	4,6
TCMDC-143187	463,8	3	4	7	5	3,67
TCMDC-143191	354,3	0	3	3	5	3,04
TCMDC-143209	378,2	2	4	8	3	3,44
TCMDC-143242	374,3	1	2	6	5	2,78
TCMDC-143254	415,3	3	3	7	4	3,99
TCMDC-143263	302,2	2	3	6	3	2,71
TCMDC-143265	407,8	1	5	6	3	2,53
TCMDC-143323	386,3	3	5	8	4	-0,04
TCMDC-143332	430,3	1	4	6	4	2,37
TCMDC-143382	378,3	3	4	5	4	2,08
TCMDC-143385	443,3	1	4	7	4	2,68
TCMDC-143408	340,3	1	4	5	4	1,81
TCMDC-143409	375,1	0	3	3	4	2,5
TCMDC-143422	279,2	0	1	2	4	3,31
TCMDC-143432	350,3	0	1	6	3	3,74
TCMDC-143454	259,6	2	2	3	3	3,38
TCMDC-143456	318,2	1	4	6	3	1,58
TCMDC-143462	434,3	2	5	7	4	3,11
TCMDC-143496	464,4	1	4	8	4	2,9
TCMDC-143513	461,8	2	4	8	5	2,4
TCMDC-143515	459,8	2	2	7	5	4,55
TCMDC-143543	460,4	0	2	6	6	4,23
TCMDC-143551	436,4	1	5	8	4	2,3
TCMDC-143592	351,7	1	1	6	3	3,9
TCMDC-143620	396,3	0	4	5	4	2,65
TCMDC-143645	363,3	3	1	7	3	4,32

MW: Molecular Weight; HBD: Hydrogen Bond donor; HBA: Hydrogen Bond Acceptor; RB: Rotable Bonds; RINGS: Number of Rings; XLOGP: XlogP (Partition coefficient score).

### Zinc-binding group assessment among lead compounds

To find ZBGs among lead compounds, a curated database of such chemotypes was first created (Table B in S1 Text). Structures were drawn using Marvin Sketcher (Chemaxon) and exported to SMILES format. This database was then imported to R and processed similarly to the MCS clustering, though instead of calculating overlapping scores between compounds, the overlapping score was determined for each compound against all ZBGs in the database. Only those compound-ZBG pairs where overlap was complete (score = 1 and, hence, ZBG completely contained in the lead compound) were counted as a match.

## Supporting information

S1 Text Supplementary information. (PDF)
