# Supplementary material for: Potent and selective inhibitors for M32 metallocarboxypeptidases identified from high-throughput screening of anti-kinetoplastid chemical boxes
Source: PLoS Negl Trop Dis. 2019 Jul 22;13(7):e0007560. doi: 10.1371/journal.pntd.0007560 (PMC6675120; doi:10.1371/journal.pntd.0007560)
Supplement: S1 Text — (PDF) [file pntd.0007560.s001.pdf]

Electronic supplementary information

Potent and selective inhibitors for M32 metallocarboxypeptidases identified from high-throughput screening of anti-kinetoplastid chemical boxes.

Emir Salas-Sarduy[1], Lionel Urán Landaburu[1], Adriana K. Carmona[2], Juan José Cazzulo[1], Fernán Agüero[1], Vanina E. Alvarez[1], Gabriela T. Niemirowicz[1*]

[1] Instituto de Investigaciones Biotecnológicas "Dr. Rodolfo Ugalde" – Universidad Nacional de San Martín – CONICET, San Martín, B1650HMP, Buenos Aires, Argentina.

[2] Departamento de Biofísica, Universidade Federal de São Paulo, São Paulo, Brazil.

***To whom correspondence should be addressed:**

Email: gniemiro@iibintech.com.ar (GTN)

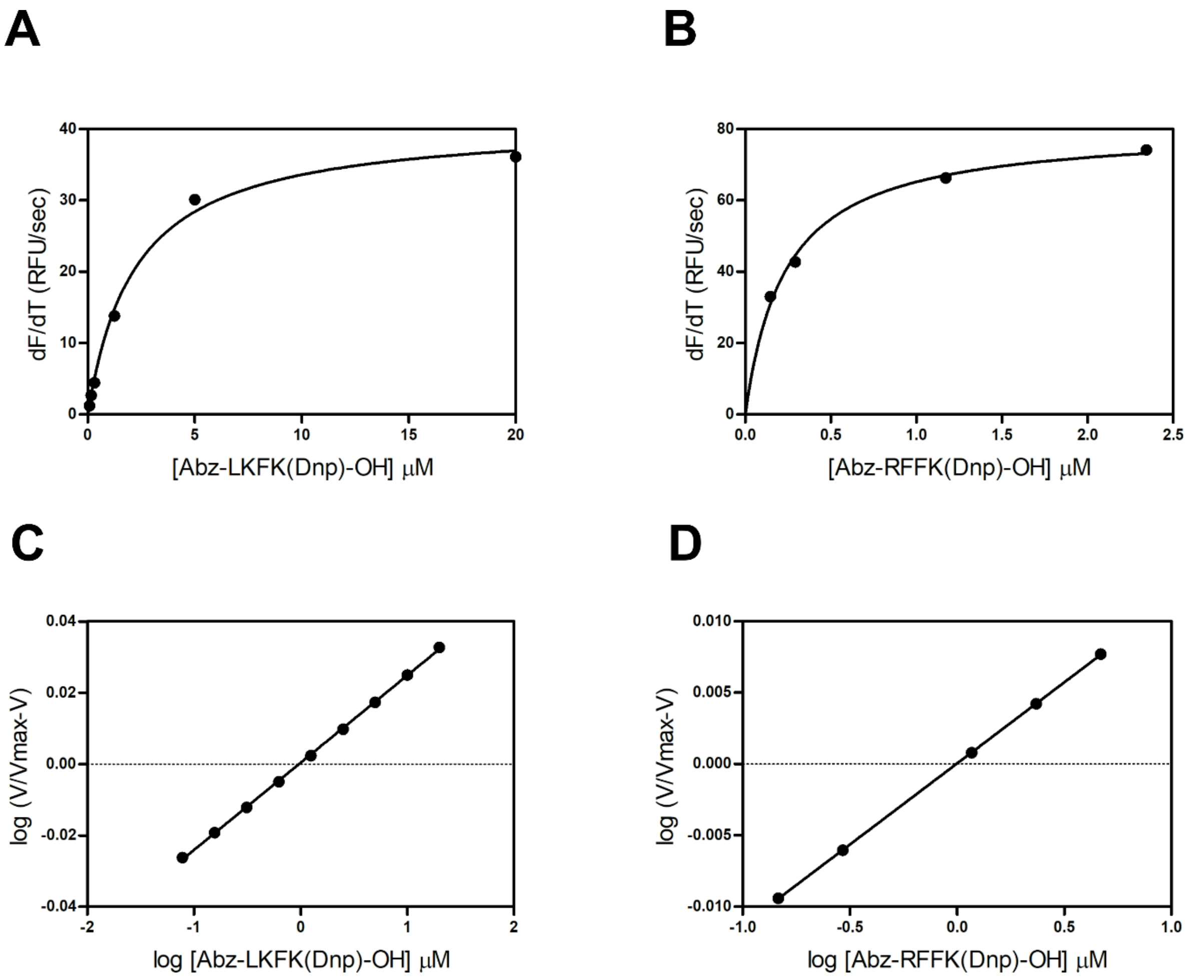

Fig A. Michaelis-Menten analysis of recombinant *Tc*MCP-1 (A) and *Tb*MCP-1 (B) acting on Abz-LKFK(Dnp)-OH and Abz-RFFK(Dnp)-OH substrates respectively. Hill plot for *Tc*MCP-1 (C) and *Tb*MCP-1 (D).

Table A. Correlation between the inhibition percentages in the primary and secondary screenings.

Compound	*Tc*MCP-1			*Tb*MCP-1		
	Primary screening Inhibition (%)	Secondary screening Inhibition (%)	2º/1º ratio	Primary screening Inhibition (%)	Secondary screening Inhibition (%)	2º/1º ratio
TCMDC-143620	82,14	96,70	1,18	45,21	96,58	2,14
TCMDC-143422	78,85	93,93	1,19	81,07	80,16	0,99
TCMDC-143456	89,09	97,67	1,10	66,24	69,00	1,04
TCMDC-143209	85,03	92,48	1,09	50,05	42,92	0,86
TCMDC-143385	42,94	80,76	1,88			
TCMDC-143172	65,01	84,56	1,30			
TCMDC-143513	65,65	70,48	1,07	56,08	42,64	0,76
TCMDC-143551	65,04	74,11	1,14	42,28	41,97	0,99
TCMDC-143462	43,06	60,29	1,40	47,90	6,29	0,13
TCMDC-143382	53,63	63,12	1,18	57,82	57,14	0,99
TCMDC-143515	54,26	55,47	1,02	40,42	50,90	1,26
TCMDC-143432	42,37	53,96	1,27			
TCMDC-143242	51,89	52,68	1,02			
TCMDC-143592	56,55	58,11	1,03			
TCMDC-143408	46,88	49,75	1,06			
TCMDC-143496	41,52	45,37	1,09			
TCMDC-143071	45,44	28,65	0,63			
TCMDC-143263	48,44	14,84	0,31			
TCMDC-143543	43,40	31,11	0,72			
TCMDC-143625	43,83	19,89	0,45	49,55	17,11	0,35
TCMDC-143105	52,36	17,76	0,34			
TCMDC-143583	77,30	-11,25	-0,15			
TCMDC-143479	42,39	13,15	0,31			
TCMDC-143172				42,44	-1,57	-0,04
TCMDC-143323				87,16	87,01	1,00
TCMDC-143409				68,91	77,95	1,13
TCMDC-143191				88,97	69,40	0,78
TCMDC-143645				42,78	54,13	1,27
TCMDC-143143				41,27	66,27	1,61
TCMDC-143332				55,90	57,63	1,03
TCMDC-143158				44,84	53,51	1,19
TCMDC-143254				45,94	35,87	0,78
TCMDC-143265				59,46	23,62	0,40
TCMDC-143454				52,66	33,23	0,63
TCMDC-143187				48,70	24,44	0,50
TCMDC-143399				43,27	8,15	0,19

TCMDC-143155	44,57	10,36	0,23
TCMDC-143159	52,96	13,07	0,25
TCMDC-143541	44,26	8,89	0,20
TCMDC-123621	44,99	-2,11	-0,05

For both MCPs, the analysis was performed using a single inhibitor concentration of 25 μM and 31,5 μM for primary and secondary screening, respectively.

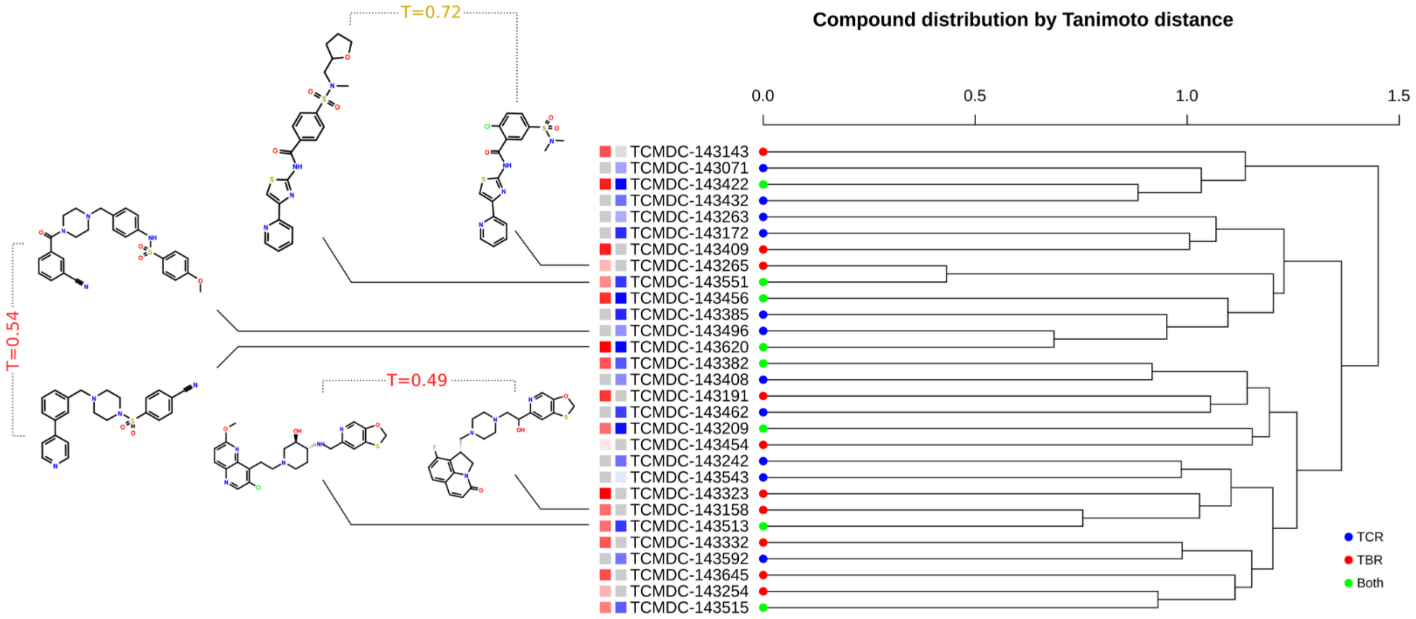

Fig B. A dendrogram representing compound clustering using Tanimoto distance between leads, and summarizing the activity distribution among tested MCPs. Squares next to the names give an idea of IC_{50} observed: the brighter the color, the lower the IC_{50}. Red squares for *Tb*MCP-1, blue squares for *Tc*MCP-1, grey squares for non-active in *Tb*MCP-1/*Tc*MCP-1, accordingly. The Tanimoto index (T) between closest pairs in the dendrogram is shown alongside their corresponding 2D structures.

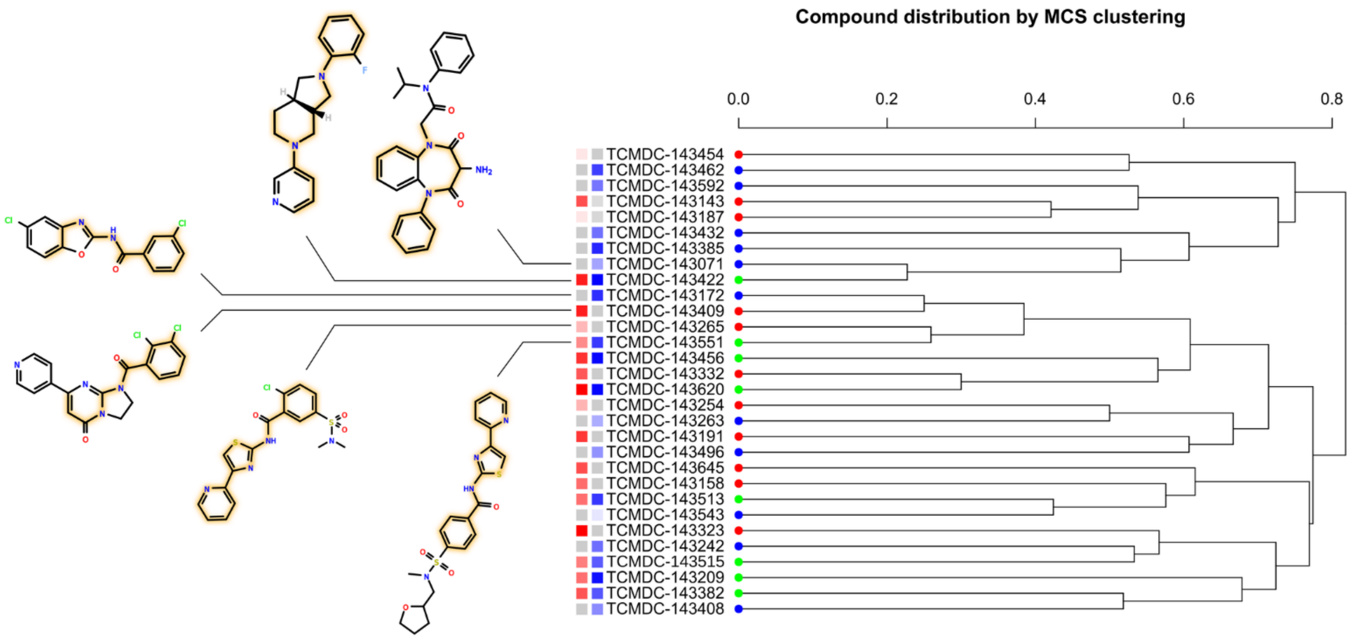

Fig C. A dendrogram representing compound clustering using MCS overlap score between leads, and summarizing the activity distribution among tested MCPs. Overlap backbones for highest rated clusters (distance < 0,25) were highlighted. Squares next to the names give an idea of IC_{50} observed: the brighter the color, the lower the IC_{50}. Red squares for *Tb*MCP-1, blue squares for *Tc*MCP-1, grey squares for non-active in *Tb*MCP-1/*Tc*MCP-1, accordingly.

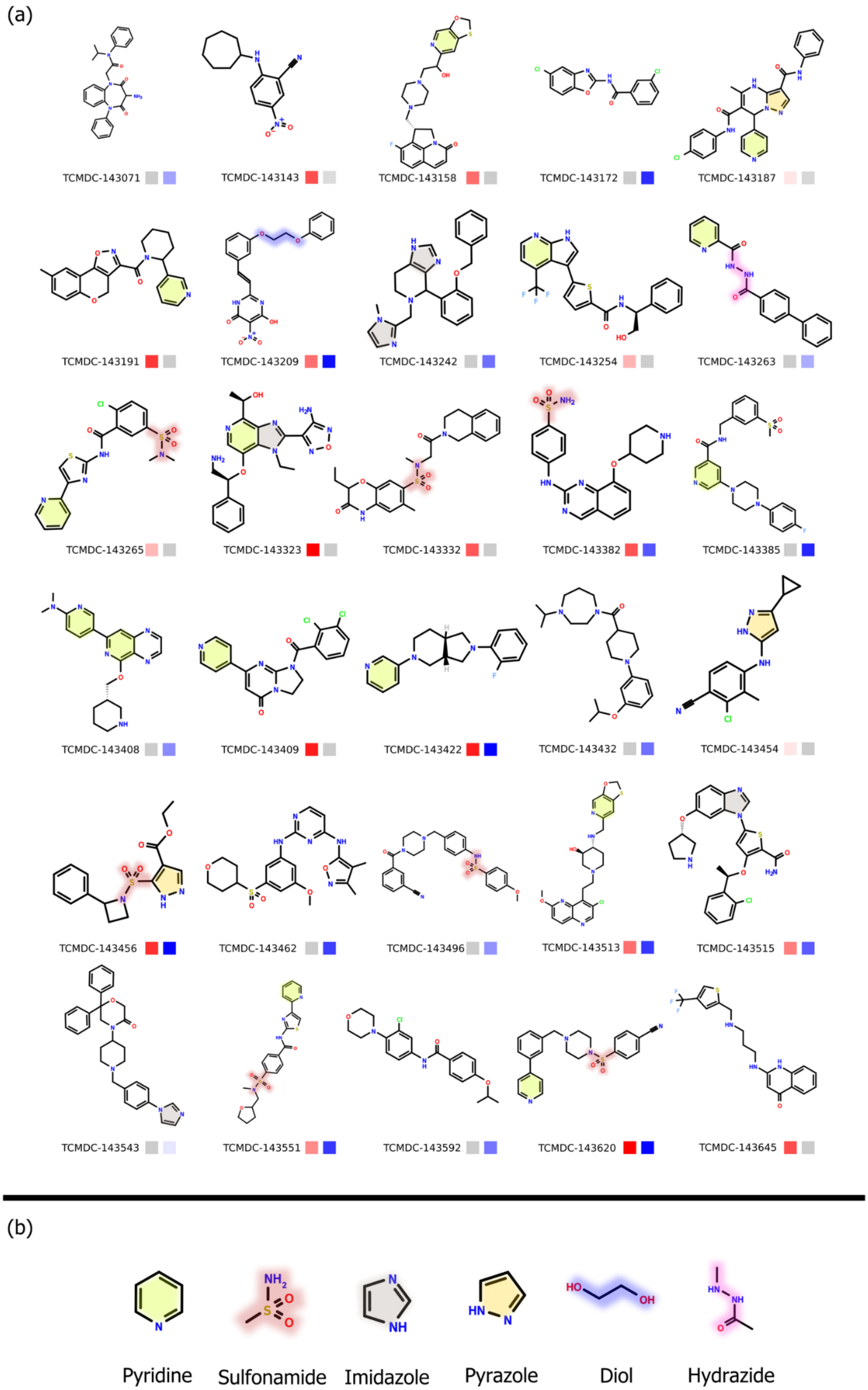
(a)
TCMDC-143071
TCMDC-143143
TCMDC-143158
TCMDC-143172
TCMDC-143187
TCMDC-143191
TCMDC-143209
TCMDC-143242
TCMDC-143254
TCMDC-143263
TCMDC-143265
TCMDC-143323
TCMDC-143332
TCMDC-143382
TCMDC-143385
TCMDC-143408
TCMDC-143409
TCMDC-143422
TCMDC-143432
TCMDC-143454
TCMDC-143456
TCMDC-143462
TCMDC-143496
TCMDC-143513
TCMDC-143515
TCMDC-143543
TCMDC-143551
TCMDC-143592
TCMDC-143620
TCMDC-143645
(b)
Pyridine
Sulfonamide
Imidazole
Pyrazole
Diol
Hydrazide

Fig D. Structure of the M32 MCPs inhibitors identified in this work. (A) All lead compounds' structures with their corresponding IC_{50}s scale. Red squares for *Tb*MCP-1, blue squares for *Tc*MCP-1, grey squares for non-active in *Tb*MCP-1/*Tc*MCP-1, accordingly. (B) Structure of the Zinc-binding groups (ZBGs) present in the identified inhibitors. ZBGs were highlighted within the compound´s structures in different colors, as indicated.

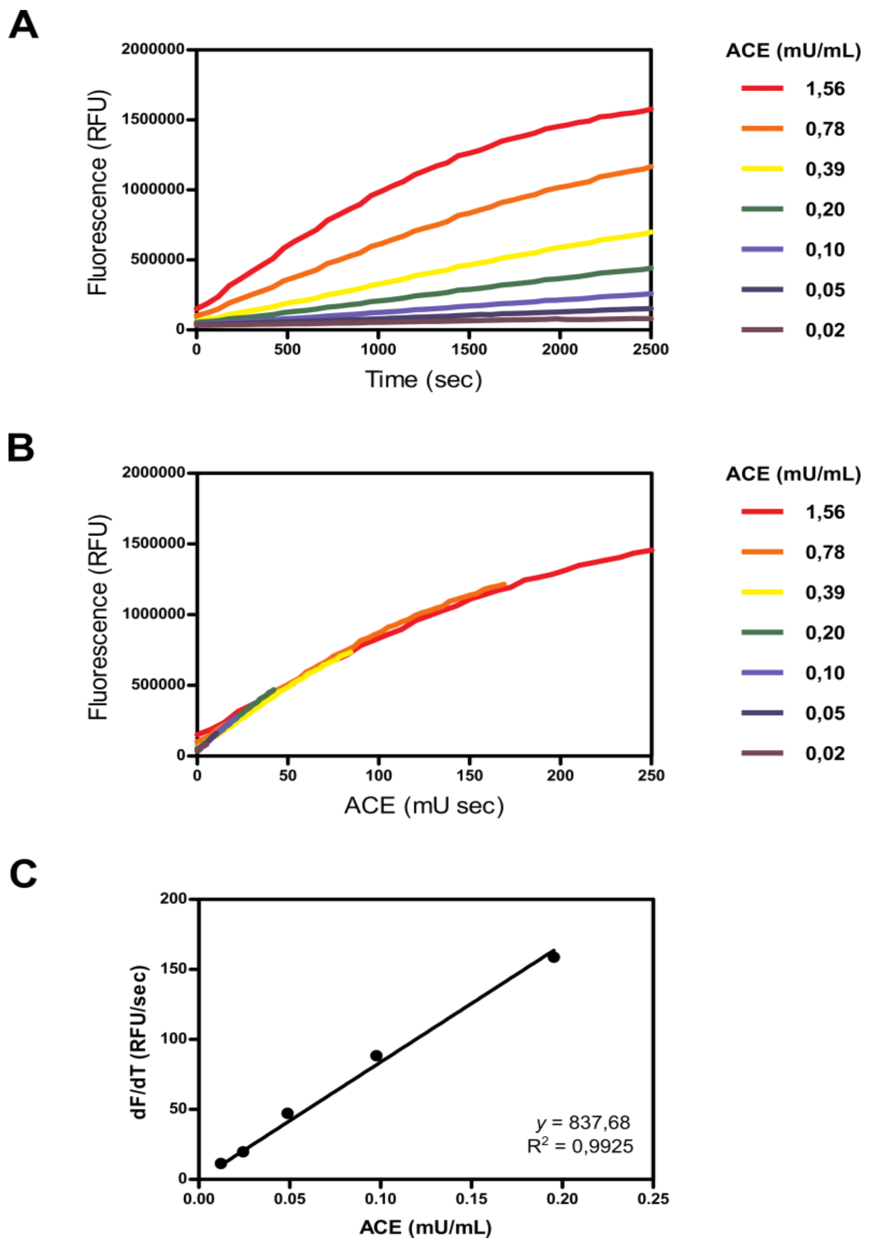

Fig E. Continuous fluorogenic assay for purified rabbit lung ACE. (A) Kinetic progression curves for different ACE concentrations at a fixed Abz-FRK(Dnp)P-OH dose (3 µM). The enzyme was assayed at 37 ºC in 0,1 M Tris-HCl pH 7,0 buffer containing 50 mM NaCl, 10 mM $ZnCl_2$ and 0,01% Triton X-100. (B) Selwyn test showed no enzyme inactivation. (C) Curve of V_0 vs. $[E]_0$. Best balance between ACE activity on Abz-FRK(Dnp)P-OH substrate (estimated as dF/dt) and the time over which the reaction displayed linear kinetics was achieved employing 0,1 mU of ACE per 1 mL reaction.

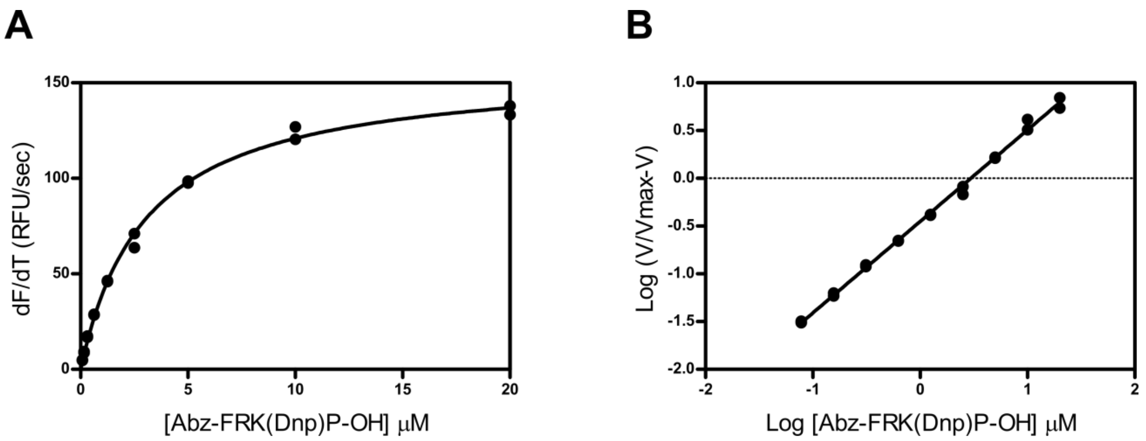

Fig F. (A) Michaelis–Menten plot for the hydrolysis of Abz-FRK(Dnp)P-OH by purified rabbit lung ACE. The enzyme was assayed at 37º C in 0,1 M Tris-HCl pH 7,0 buffer containing 50 mM NaCl, 10 mM $ZnCl_2$ and 0,01% Triton X-100. In these conditions the K_M value was 3,04 ± 0,15 µM. (B) The Hill plot for ACE (Hill coefficient 0,9581± 0,0133).

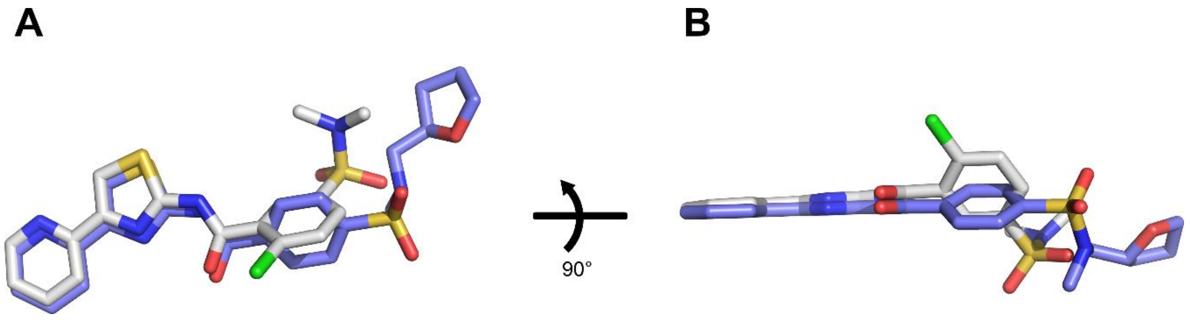

Fig G. Structural alignment of TCMDC-143265 and TCMDC-143551. (A) Top view. (B) Lateral view. A significant part of both molecules (pyridine and thiazole rings, amidyl group) is identical and adopts the same spatial conformation. In contrast, both structures differ significantly around the benzamide ring and sulfonamide substituents.

Table B. Curated database of Zinc-binding groups.

ID	SMILES
CMP1	C1=CC=NC=C1
CMP2	C1CNCCNCCCNCCNC1
CMP3	C1CNCCNCCN1
CMP4	C1CNCCNCCNCCN1
CMP5	CC1=CC2=CC=CC(O)=C2N=C1
CMP6	CC1CCCCN(O)C1=O
CMP7	CC1CCCN(O)C1=O
CMP8	CC1=C(O)C(=O)C=CO1
CMP9	CC1=C(O)C(=S)C=CO1
CMP10	C(C1=NC2=CC=CC=C2N1)C1=NC2=C(N1)C=CC=C2
CMP11	CC1NCCCN(O)C1=O
CMP12	CCC(O)=O
CMP13	CC(=O)NNS(C)(=O)=O
CMP14	CC(=O)NO
CMP15	CC(S)C(N)=O
CMP16	CN1C=CC=C(O)C1=O
CMP17	CN1C=CC=C(O)C1=S
CMP18	CN1C=CC(=S)C(O)=C1C
CMP19	CNC1=C(N(C)O)C(=S)C1=O
CMP20	CNC1=C(N(O)[*])C(=O)C1=O
CMP21	C(NCC1=NC=CC=C1)C1=CC=CC=N1
CMP22	CN(C)C(O)=O
CMP23	CNC(=O)NO
CMP24	CNC(=O)P(O)(O)=O
CMP25	CNNC(C)=O
CMP26	COC(=O)CN(O)C(C)=O
CMP27	CSCCS
CMP28	CS(C)=O
CMP29	CS(N)(=O)=O
CMP30	N1C=CC=N1
CMP31	N1C=CN=C1
CMP32	N1C=NN=C1
CMP33	NC(N)=O
CMP34	NC(=O)CCN(O)=O
CMP35	OC1CCC2NNC(=O)C2C1
CMP36	OC1=CC=CNC1=O
CMP37	O=C1CC(=O)NC(=O)N1
CMP38	O=C1CC(=O)NC(=S)N1
CMP39	OC1=COC=CC1=S
CMP40	OC1NC(=O)NC=C1
CMP41	OC[C@@H]1NC([*])O[C@H]1C=C
CMP42	OCCO
CMP43	OC(=O)C1=CC=CC=N1
CMP44	OC(=O)C1=CC=CC(=N1)C(O)=O
CMP45	OC(=O)CNCC(O)=O
CMP46	ON1C=CC=CC1=O
CMP47	O[N]1=CC=CC=C1S
CMP48	ON1C=CC=CC1=S

For each chemotype, identifier and SMILES code are indicated.
